# Effect of Sintering on In Vivo Biological Performance of Chemically Deproteinized Bovine Hydroxyapatite

**DOI:** 10.3390/ma12233946

**Published:** 2019-11-28

**Authors:** Bruno De Carvalho, Eric Rompen, Geoffrey Lecloux, Peter Schupbach, Emilie Dory, Jean-François Art, France Lambert

**Affiliations:** 1 Department of Periodontology and Oral Surgery, University of Liège, 4000 Liège, Belgium; bruno.decarvalho@chuliege.be (B.D.C.); eric.rompen@wishbone-biotech.com (E.R.); geoffrey.lecloux@chuliege.be (G.L.); 2 Adjunct Professor, Augusta University, Augusta, GA 1120, USA; pmschupbach@mac.com; 3 Department of Biomaterials, WishBone SA, 4000, Liège, Belgium; emilie.dory@wishbone-biotech.com (E.D.); jean-francois.art@wishbone-biotech.com (J.-F.A.)

**Keywords:** bone regeneraton, hydroxyapatite, osteoconduction, surface microtopograraphy, sintering

## Abstract

The influence of the manufacturing process on physicochemical properties and biological performance of xenogenic biomaterials has been extensively studied, but its quantification on bone-to-material contact remains poorly investigated. The aim of this study was to investigate the effect of different heat treatments of an experimental chemically-deproteinized bovine hydroxyapatite in vivo in terms of new bone formation and osteoconductivity. Protein-free hydroxyapatite from bovine origin was produced under sub-critical conditions and then either sintered at 820 °C or 1200 °C. Structural and morphological properties were assessed by scanning electron microscopy (SEM), measurement of surface area and X-ray diffractometry (XRD). The materials were then implanted in standardized alveolar bone defects in minipigs and histomorphometric evaluations were performed using non-decalcified sections. Marked topographical differences were observed by SEM analysis. As the sintering temperature of the experimental material increased, the surface area significantly decreased while crystallite size increased. In vivo samples showed that the highly sintered BHA presented a significantly lower percentage of newly formed bone than the unheated one (*p* = 0.009). In addition, the percentage of bone-to-material contact (BMC) was significantly lowered in the highly sintered group when compared to the unsintered (*p* = 0.01) and 820 °C sintered (*p* = 0.02) groups. Non-sintered or sintered at 820 °C BHA seems to maintain a certain surface roughness allowing better bone regeneration and BMC. On the contrary, sintering of BHA at 1200 °C has an effect on its morphological and structural characteristics and significantly modify its biological performance (osteoconductivity) and crystallinity.

## 1. Introduction

Alveolar bone regeneration procedures are often performed to treat bone defects in order to replace missing teeth with dental implants in a multitude of situations in dentistry [1,2]. If autogenous bone grafts are still considered as the “gold standard” due to their osteogenic properties, their use is associated with several limitations, including limited bone supply, high resorption rates [3] and morbidity at the donor site, affecting patient compliance [4,5]. Therefore, biomaterials have become an alternative increasingly used in oral surgery, implantology and periodontology as bone void fillers allowing bone in-growth, acceleration of bone remodeling and osteoconductive structural guidance [6,7].

Calcium-phosphate-based (CaP) materials have received a lot of attention due to their chemical similarity to bone, but also due to their excellent biocompatibility [8,9]. Natural and synthetic CaP materials such as hydroxyapatite (HA) and tricalcium phosphate (TCP) are the most commonly used biomaterials for alveolar bone regeneration [10]. TCP materials are osteoconductive and support bone growth by delivering calcium and phosphate ions in situ [11,12]; however, their fast biodegradation rate may not be the most appropriate to maintain bone volume overtime, which is indicated in cases of implant site development [12,13,14]. By contrast, HA which is also highly osteoconductive and similar to the mineral composition of natural bone, has the advantage of being more stable in body fluids [10,14,15]. Natural HA from bovine origin (bovine hydroxyapatites, BHA) remains the most used material in oral surgery, compared to synthetic HA, exhibiting physicochemical properties close to natural bone such as surface structure, morphology and microporosities (1–50 μm) [16,17]. According to some authors, these characteristics may influence the osteoconductivity of the materials and, therefore, their clinical performance [18,19,20,21]. Additionally, their slowly resorbable feature is of great interest in the field of dentistry and maxillofacial surgery, as one of the goals of bone regenerative treatment is to maintain the volume over time [22]. 

For safety reasons, xenogenic materials of bovine origin need to be completely deproteinized through thermal or chemical strategies, to eliminate the risk of transmission of bacteria, virus or prion particles [21,23,24,25]. One easy and cheap process to deproteinize BHA is sintering at temperatures higher than 1000 °C, ensuring the complete removal of the organic compounds and improving mechanical properties (density, grain size, compressive, flexural and torsional strength) [26,27]. Commercially available xenogenic HA are manufactured with sintering temperatures up to 1300 °C. Despite the effect of sintering temperature on the physical and chemical characteristics of HA being well documented [16,26,28,29,30,31,32], its quantification on bone to material contact is poorly investigated. Thus, it is essential for the clinician to know what is the biological impact of this particular manufacturing process. Sintering hydroxyapatite has a significant impact on the surface properties; involving surface roughness, on microstructure including the crystallinity and microporosity [33,34,35] and may, therefore, influence the in vivo biological performance of the biomaterial [20,36,37]. Structural characteristics, such as microporosity, surface roughness, and specific surface area were shown to be of great importance in promoting protein adsorption on the material surface and capillary effect, and would consequently influence cell adhesion, proliferation, and bone tissue growth [17,38,39,40,41,42]. The physical characteristics seem to be a key feature, significantly influencing bone-regeneration outcomes [39]. 

The primary objective of this study was to investigate the effect of different sintering temperatures of chemically-deproteinized bovine hydroxyapatite in vivo in terms of biological performance (new bone formation and osteoconductivity). The secondary objective was to correlate the in vivo results with the structural and morphological characterization of the studied biomaterials.

## 2. Materials and Methods

### 2.1. Experimental Biomaterials

Three experimental hydroxyapatite xenografts from bovine origin were tested in the present study for physicochemical characterization and biological evaluations. The three groups were chemically deproteinized, in which the complete elimination of organic residues was performed under sub-critical conditions and a continuous flow of a basic solution following the patented extraction method developed by Wishbone SA (WO 2015/049336 A1) [44]. After elimination of the organic compounds, the two BHA samples were sintered under atmospheric conditions for 30 min at 820 °C (HA820) or 1200 °C (HA1200) while the third one was not sintered (HAN). The temperature of 820 °C was chosen based on mechanical tests and scanning electronic microscopy (SEM) analysis regarding the physicochemical characteristics. In both cases, the sintering was performed as follows: the material was introduced in the furnace once the temperature had reached 800 °C; then the furnace heated until it achieved the sintering temperature (820 or 1200 °C); the material was sintered during 30 min at the chosen temperature; after 30 min, the furnace was shut down and the material was retrieved once the furnace had naturally reached room temperature again. All sintering parameters (time and temperature) were controlled by the internal controller of the furnace. The samples, HAN, HA820 and HA1200, were further sterilized in a drying oven at 120 °C. The material was not rinsed between sintering and sterilization. For all samples, small granules (particles size: 250–1000 µm) were used 

### 2.2. Animals

Yucatan mini-pigs were used for this study because their bone turns over in a similar manner to that of humans. Moreover, these animals are used in dental research due to similarities with humans in tooth development eruption time and sequence of tooth eruption [45]. The animals were 18 months old with a weight between 50 to 60 kg and presenting a complete eruption of the permanent teeth. All experimental procedures and protocols used in this investigation were reviewed and approved by the CIRE platform agreement number A37-175-4, Tours, France. The Animal Research Reporting of In Vivo Experiments (ARRIVE) guidelines as well as national and European legislation were carefully followed. 

### 2.3. Study Design

**Figure 1:**
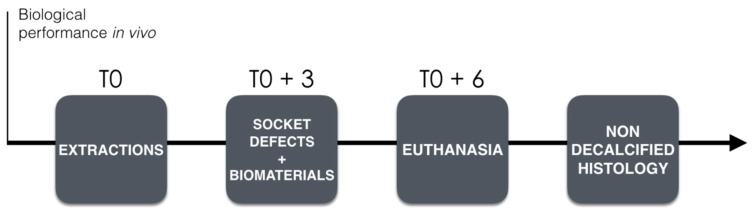
Study time line on biological performance.

Before implantation, the raw experimental biomaterials were characterized by scanning electronic microscopy (SEM) for qualitative analysis, measurement of the surface specific area (BET) and by X-ray diffractometry (XRD) to identify the crystalline phases and measurement of the crystallite size. The biological performance of the biomaterials was then evaluated in vivo in a mini-pig model (Figure 1). Dental extractions (T0) were performed in the maxillae of 5 mini-pigs. After a healing period of 3 months, six standardized bone socket defects were created per animal (3 per hemi-maxilla) and then filled with each of the three experimental biomaterials. A total of 10 samples per condition were considered for analyses. The software GPower was used to calculate the total sample size for 3 groups of biomaterial considering a statistical power of 0.8 and an effect size of 0.6 obtaining a total sample size of 30. Given the number of 6 implantation sites per animal, a total of 5 mini-pigs were chosen for this study. Euthanasia was performed after a healing period of 3 months, and the samples were subjected to non-decalcified histological analysis and histomorphometry.

### 2.4. Scanning Electronic Microscopy (SEM) Analysis 

The three biomaterials were mounted on a glass slide double-sided carbone tape with platinum (20 nm) coated in a Balzers SCD030 sputtering unit. The surface characteristics were then captured using a scanning electron microscope ESEM-FEG XL30 (Philips Electron Optics, Hillsboro, OR, USA) at magnification levels of ×20,000, ×5000, and ×50 for descriptive analysis. 

### 2.5. Brunauer–Emmett–Teller (BET) Specific Surface Area Analysis 

The specific surface area of each product was measured by N_2_ adsorption according to the Brunauer–Emmett–Teller (BET) method. Nitrogen adsorption-desorption isotherms were registered on a TriStar 3000 equipment (Micromeritics, Norcross, GA, USA). Prior to the measurements, the samples were degassed overnight under vacuum at 120 °C on a VacPrep 061 (Micromeritics, Norcross, GA, USA). For each product, the measurement was repeated two times on three independent samples.

### 2.6. X-ray Diffraction (XRD) Analysis

Powder X-ray diffraction (XRD) was performed in Bragg–Brentano geometry using a Bruker D8 Twin-Twin diffractometer with Cu Kα radiation (Billerica, MA, USA). The three products were ground into fine powders using an agate mortar. Three independent diffractograms were collected on each product in the 3°–70° 2θ-range with a 0.02° step size. The TOPAS software [46] was used to estimate the crystallite size; structure parameters for the hydroxyapatite phase were taken from the PDF 01-085-5086 reference (ICCD PDF4+ database) and the fundamental parameters approach was used to model the instrumental contribution to the reflection profiles. The crystallite size contribution to the peak profiles was modeled as lorentzian and convoluted with the instrumental contribution; the crystallite size (CS) and the integral breadth β of the lorentzian (= area under the diffraction peak divided by maximum height of the peak) are related through the equation CS = λ / (β cos θ) where λ is the Cu K_alpha1_ wavelength and θ is half of the 2θ angle [47].

### 2.7. Surgical Procedure

**Figure 2:**
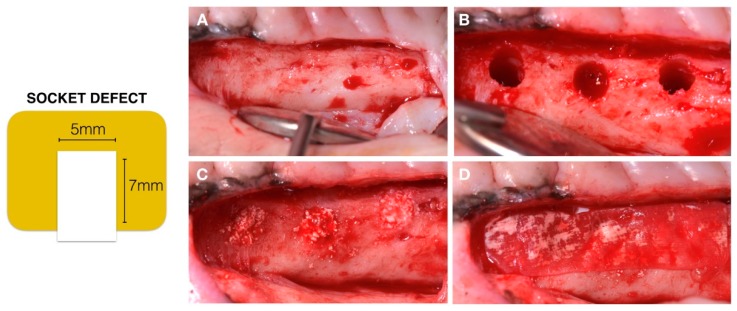
(**A**) Maxillae alveolar ridge after 3 months’ extraction; (**B**) Socket defect; (**C**) Sockets filled with the 3 experimental biomaterials; (**D**) Collagen membrane covering socket defects.

All the animals were sedated with xylazine (Ronpums, 1 mg/kg, Bayer, Munchen, Germany) and imalgene 1000 (Imalgene^®^, 15 mg/kg, Merial Laboratory, Lyon, France). During the surgery, the animals inhaled O_2_, were surveyed with an electrocardiogram and were maintained with a perfusion of imalgene 1000 (Imalgene^®^, 15 mg/kg, Merial Laboratory, Lyon, France) in the saline. One hour before surgery, an injection of rimadyl^®^ (carprofen, Kalamazoo, Michigan, USA) 1.5 mg/kg was performed. All the surgical procedures were performed under aseptic conditions. After disinfection of the surgical field with povidone iodine 10% (Iso-Betadine^®^ titratable iodine, Meda Manufacturing, Merignac, France), a local anesthesia was performed at the maxillae (4% articaine hydrochloride with epinephrine 1:100,000 Septanest Sp; Septodont Inc. Créteil, Saint-Maur-des-Fossés, France). An intrasulcular incision and a full thickness flap were made from the canine to the second molar in each hemi arcade. The extractions of the four pre-molars and first molar were made carefully by a technique of root separation. After the extractions, the surgical sites were closed with resorbable sutures (Vicryl 4-0, Ethicon GmbH, Norderstedt, Germany). The following post-surgical treatments and medication were administrated: antibiotic coverage of Amoxiciline for 5 days (Streptocillin^®^ Vet, Boehringer Ingelheim, Copenhague, Danemark), analgesics (buprenorphine, Temgesic Schering-Plough, Brussels, Belgium) and daily mouth spray of chlorhexidine gluconate 0.2%, (Corsodyl^®,^ GSK, Brentford, UK). After a healing period of 3 months, socket bone defects were created as follows. A supra crestal incision was performed and mucoperiosteal flaps were elevated to expose the right and left edentulous crest in the maxilla (Figure 2A). Three socket defects of 7 mm in depth and 5 mm in diameter were created in each hemi arcade (Figure 2B). The 3 biomaterials were randomly allocated in the standardized socket created at each hemi arcade and a collagen membrane (creos xenoprotect, Nobel Biocare AB, Göteborg, Sweden) was sized to cover the 3 bone defects in order to prevent the internal growing of soft tissue (Figure 2C,D). The mucoperiosteal flaps were sutured with non-resorbable sutures (polypropylene 4-0 FS-2; Ethicon, Edinburgh, UK). 

### 2.8. Euthanasia

After a healing period of 6 months (T0+6), euthanasia was performed with an overdose of sodium pentobarbital at 3%. The maxillary blocs were resected with an oscillating saw autopsy and the specimens containing the biomaterial sites and surrounding hard and soft tissues were placed in a 4% formaldehyde solution for sample fixation. 

## 3. Histology 

Histological evaluation was performed on undecalcified polymethylmethacrylate (PMMA) sections. Dehydration was performed using an ascending concentration ethanol bath for 24 h: 1 × 70°, 1 × 80°, 2 × 95°and 3 × 100°, and then by soaking for 24 h in acetone. Subsequently, samples were impregnated with methyl methacrylate for 48 h at 20 °C with a refreshment of the medium. Finally, they were embedded in PMMA at 4 °C for 4 days. The sections were stained using methylene blue (1%) for 90 s and basic fuchsin (0.3%) for 25s for a descriptive analysis. Sections were prepared using the cutting-grinding technique of hard tissues according to Donath and Breuner, 1982 [48].

### 3.1. Histomorphometry

**Figure 3:**
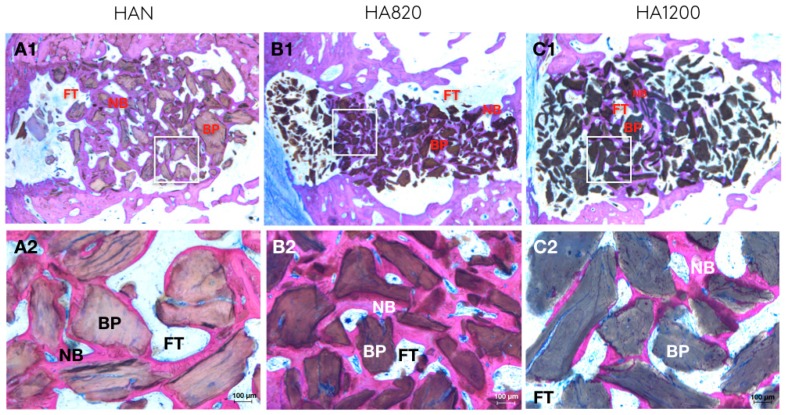
Descriptive histology after 3 months. BP, bone particle; NB, newly formed bone; FT, fibrous connective tissue.

**Figure 4:**
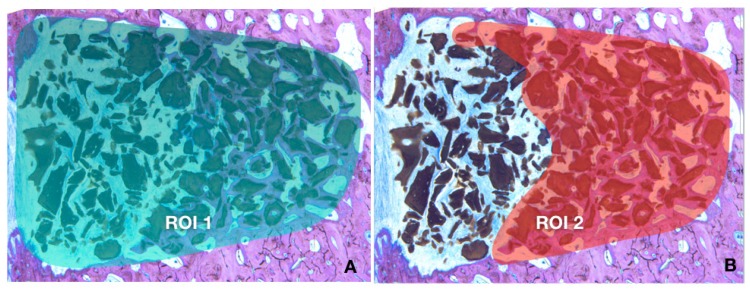
(**A**) Defect area (ROI1); (**B**) Regenerated area (ROI2).

The resulting sections (Figure 3) were digitalized using a Microscope (Leica 205A stereomicroscope Wetzlar Germany) in order to perform quantitative measurements of mineralized bone and biomaterial using image analysis software (ImageJ, National Institute of Health, Bethesda, MD, USA). The following parameters were calculated:

- Defect area: defined as the total area of the defect (Region Of Interest, ROI1) Figure 4A
- Regenerated area: defined as the defected area colonized by newly formed bone (ROI2) Figure 4B
- % of regeneration: proportion of ROI2/ROI1.
- % of newly formed bone, % of biomaterial, % of soft tissue within the overall defect area (ROI1)
- The osteoconductivity characterized by the bone to material contact (BMC) defined by the percentage of particles perimeter in contact with newly formed bone within the regenerated area (ROI2).

**Figure 5:**
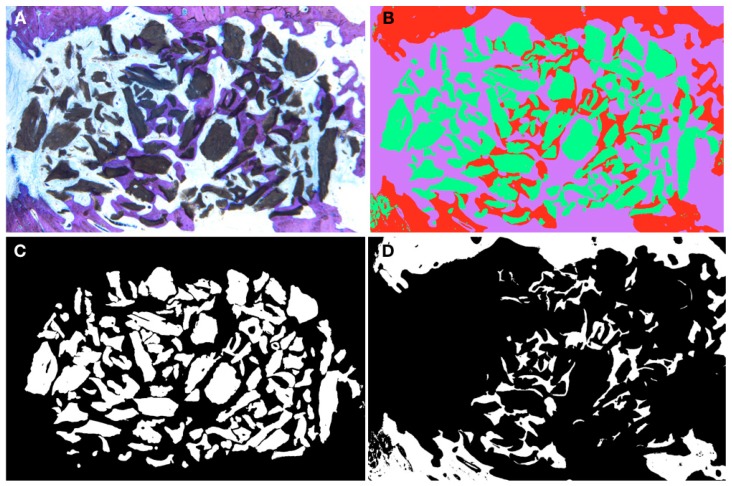
Histomorphometry segmentation steps; (**A**) Example of histological cut HA820; (**B**) Color segmentation using ImageJ; (**C**) Binary image isolating all the particles of bovine hydroxyapatites (BHA); (**D**) Binary image isolating the bone content.

All images were segmented in 3 colors, red, green and purple, identifying respectively bone, biomaterial and soft tissue. The segmented images (Figure 5B) were then transformed into binary images allowing the individualization of each of the segmentations (Figure 5C). The software calculated the % of pixels present in each image. For the BMC, the 2 binary images (bone and biomaterial) were fused using a macrocode, drawing automatically the contact between the 2 segments. The total length of the contact lines was then measured and divided by the total perimeter of the particles contained in the ROI2.

### 3.2. Statistical Analysis

Results were presented as means and standard deviation (SD). A general linear mixed model (GLMM) was used for the comparisons of the parameters between the 3 groups (HAN, HA820 and HA1200) six measurements were done in the same pig. The Scheffé test was used to compare temperatures two by two. The results are considered to be significant at the 5% level (p < 0.05). Statistical analyses were performed using SAS software version 9.4 (SAS Institute, Cary, NC, USA).

## 4. Results

### 4.1. Study Design

During the healing period, one of the mini-pigs showed signs of paralysis of the hind legs and early euthanasia was performed. Therefore, a total of 24 bone defects and 8 samples per condition were available from the 4 remaining mini-pigs. All samples were assessed quantitatively and descriptively. 

### 4.2. SEM Characterization

**Figure 6:**
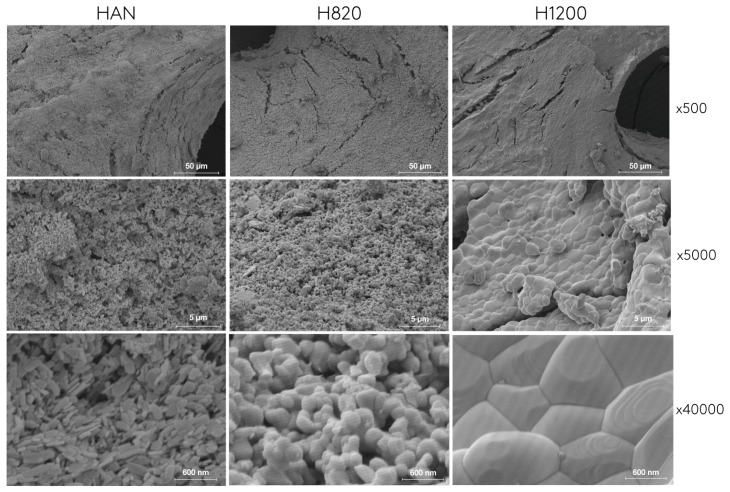
Scanning electronic micrographs (SEM) of the studied biomaterials at different magnifications: HAN: non-sintered HA; HA820: 820 °C sintered HA; HA1200: 1200 °C sintered HA.

The SEM analysis provided a graphic insight of the HA structure with respect to porosity. SEM images of HAN, HA820 and HA1200 are displayed in Figure 6. The bone-like macro-architecture with macropores (>50 μm) was visible in all samples. When looking at higher magnification, micropores could be observed in HAN and HA820 samples. HAN seems to preserve its surface roughness architecture while H820 changed into grain-like architecture presenting a rougher surface than HA1200 that presents a rather smooth surface and very few micro porosities. 

### 4.3. Surface Area, Pore Size and Pore Volume Analysis

Surface area, pore size and volume are presented in Table 1. BET surface area analysis was carried out on all samples. From the average of two independent BET isotherms, the surface areas of HAN, HA820 and HA1200 were ~36, ~4.2 and ~0.27 m^2^/g respectively, which showed that surface area substantially decreased with sintering. Barrett-Joyner-Halenda (BJH) pore size and volume analysis was also carried out. Both pore size and volume decreased with sintering temperature, with HAN, HA820 and HA1200 showing respective average pore sizes of ~10, ~5.2 and ~4.5 nm, and respective total pore volumes of ~0.13, ~0.007 and ~0.003 cm^3^/g.

### 4.4. XRD Analysis

**Figure 7:**
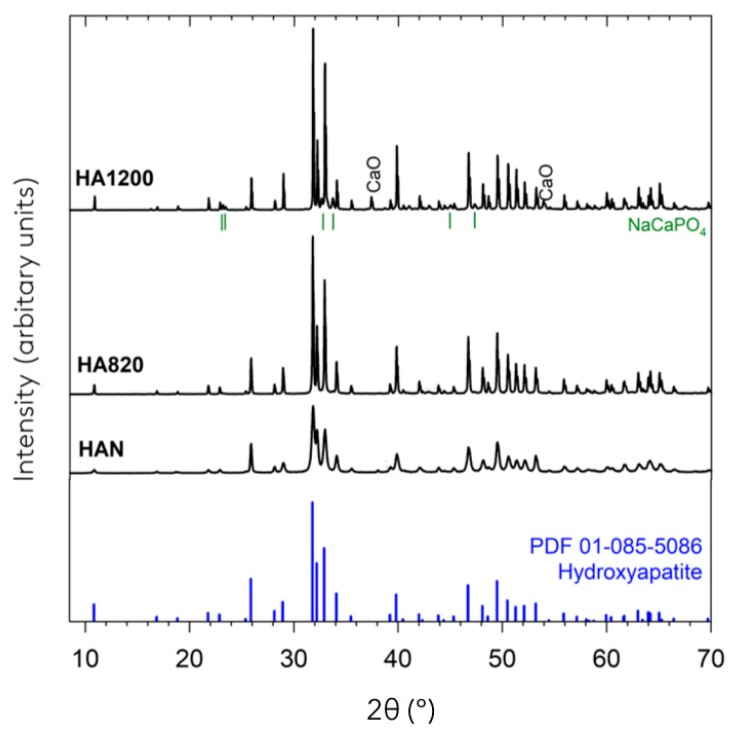
Comparison of the X-ray diffraction patterns of HAN, HA820, HA1200 with the reference spectra of hydroxyapatite.

The powder XRD patterns of representative samples are presented in Figure 7. For HAN, all observed peaks could be indexed to the formation of hydroxyapatite, Ca_10_(PO4)_6_(OH)_2_ (*P*6_3_*m*; *a* = 9.424 Å, *b* = 9.424 Å, c = 6.879 Å, α = 90°, β = 90°, γ = 120°) [49]. For HA820 and HA1200, the majority of peaks could also be indexed to the formation of hydroxyapatite; however two other crystalline phases were detected. In HA820, a small broad peak at ~43° 2Θ was observed, which may correspond to the formation of NaCaPO_4_ (*Pn*2_1_*a*; *a* = 20.397 Å, *b* = 5.412 Å, *c* = 9.161 Å, α = 90°, β = 90°, γ = 90°) [50]. In HA1200, several additional peaks corresponding to the presence of NaCaPO_4_ were observed, and one additional peak at ~37.5° 2Θ that may correspond to the formation of CaO (*Fm*$\bar{3}$*m*; *a* = 4.8152 Å, *b* = 4.8152 Å, *c* = 4.8152 Å, α = 90°, β = 90°, γ = 90°) [51]. With an increase in sintering temperature, peaks in the majority hydroxyapatite phase became sharper and narrower, which was a result of an increase in the average crystallite size. When fitting the reflection with a model taking into account crystallite size and instrumental contributions, the average crystallite size increases from 29 nm in HAN, to ~120 nm in HA820 to ~230 nm in HA1200.

### 4.5. Descriptive Histology

After 3 months, newly formed bone was observed in the defect of all samples, mainly in the apical 2/3 and adjacent to the lateral bone walls of the artificially created sockets (Figure 3). The typical structure of a newly formed bone is observed, with the presence of osteocytes entrapped by lamellar bone. Some chains of osteoblasts are also observed close to the newly formed bone as well as some blood vessels, with no visible differences between samples. No signs of inflammation were visible in any of the sections. Generally, samples of HA1200 showed less newly formed bone with more predominance of soft tissue between particles. In all groups it was possible to observe the particles in close contact with newly formed bone; however, this was predominantly observed in HAN and HA820.

### 4.6. Quantitative Histomorphometric Analysis

**Figure 8:**
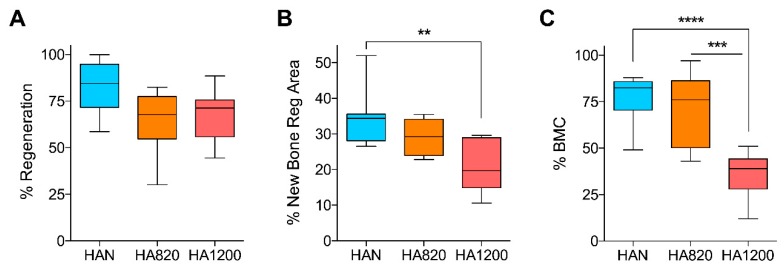
(**A**) % of Regeneration; (**B**) % of newly formed bone within the ROI2; (**C**) % of Bone to material contact; ** = p < 0.01; *** = p < 0.001; **** = p < 0.0001.

The % of regeneration (Figure 8A) was homogeneous in all groups with no significant differences (*p* = 0.10). In terms of % newly bone formation in the regenerated area (Figure 8B), there were significant differences between HAN and HA1200 (*p* = 0.009), although no significant differences were detected between HA820 and HAN (*p* = 0.119) and between HA820 and HA1200 (*p* = 0.115). The osteoconductivity (bone to material contact) (Figure 8C) was also significantly higher in the groups HAN and HA820 compared to HA1200 (HAN vs. HA1200 *p* = 0.0192; HA820 vs. HA1200 *p* = 0.0234). There was no significant difference between HAN and HA820 (*p* = 0.8876). 

### 4.7. Correlation Between Structural Characterization and Biological Performance

For all samples, a positive correlation was observed between higher surface area values and % of newly bone (*p* = 0.001) and % of BMC (*p* = 0.04). By contrast, a negative correlation was observed between higher crystallite size values and percentage of newly formed bone (*p* = 0.0002) and percentage of BMC (*p* = 0.001).

## 5. Discussion

The present study emphasized that the sintering process at high temperature modulates not only the structure but also the biological performance of chemically-deproteinized BHA.

The sintering process can result in increased crystallinity, appearance of new crystalline phases, decreased porosity and changes in the surface morphology. These characteristics may influence the performance results [26,42] Nevertheless, the sintering temperature remains an important parameter for the improvement of mechanical properties of BHA [26,27,52].

The analysis of the hydroxyapatite structure by XRD revealed that the main crystalline phase present in all samples is hydroxyapatite. This proves that the chemical deproteinization process used here does not deteriorate the main crystalline phase initially present in bovine bones. The peaks corresponding to hydroxyapatite appear sharper when the sintering temperature increases, which can be interpreted as an increase in crystallite size and is considered a sign of high crystallinity. The crystallinity of a biomaterial is important as it influences its dissolution; therefore, high crystallinity materials display lower biodegradation rates and better volume stability overtime [53,54,55,56].

Additional traces of NaCaPO_4_ and CaO were also detected in HA1200. The formation of CaO at 1200 °C could result from the decomposition of the carbonate content initially present in natural bone as already observed in other studies [57].

SEM analysis revealed that, from a descriptive point of view, the unsintered particles preserved their surface roughness architecture while the particles sintered at 820 °C displayed a grain-like architecture. By contrast, the particles subjected to 1200 °C suffered from structural changes on the surface morphology, presenting a smoother surface and fewer micro porosities compared to the two other BHA which can be correlated to the fusion of crystals as described by Patel, 2001 [58]. Looking at the porosity using BET analysis, the surface area decreased sharply from 36 to 4.2 m^2^/g when the particles were sintered at 820 °C, and even to 0.27 m^2^/g when sintered at 1200 °C. The specific surface area has been often mentioned in the literature as a significant parameter for bone regeneration [37,54,58,59,60]. It is indeed believed that a high specific surface area promotes extracellular matrix protein and cell adsorption, and thus favors bone formation [38]. According to the present results, the in vivo behavior of biomaterials seems to correlate better to their surface morphology than to their specific surface area. When the sample is sintered at low temperature (820 °C) the biological performance was not altered compared to the non-sintered sample. However, a clear change of the biological behavior was observed when the material was sintered at higher temperature (1200 °C) and the surface became smoother. Smooth surfaces are less expected to conduct cell colonization [61]. Indeed, the histomorphometric results of this study showed that the percentage of newly formed bone and the osteoconductivity (bone-to-material contact, BMC) was equivalent for low and non-sintered hydroxyapatites, despite their significant difference in specific surface area while the BMC was significantly lower for the HA sintered at 1200 °C. Therefore, it seems that the surface morphology of BHA (rather than specific surface area) is a key factor that should be considered when assessing their biological performance. These results seem in accordance with the literature about dental implants as the positive correlation of the roughness of implants and osteoconduction has been well described in several systematic reviews [62,63]. Nevertheless, it should be emphasized that one of the limitations of the present study was the lack of quantitative measurement of the micro-roughness of the experimental materials due to the complexity of this technique on particulate materials such as BHA. Another limitation of this study is that no comparison was made to BHA that did not undergo chemical deproteinization before sintering, which is how the commercially available sintered BHA products are currently made. Such variations in the manufacturing process may affect the physicochemical properties and provide different in vivo performance. Also, the specific production process steps might influence the BHA structure and consequently the clinical results. Nevertheless, good osteoconductivity and clinical results of sintered bovine bone has been reported many times [31,32].

The present study is one of the few taking into account the BMC to assess the biological performance of regenerative biomaterials since it is intended that each particle should be able to osteointegrate and have a high contact interface with newly formed bone, forming a tight bone–material network, which plays an important role in the subsequent implant placement and survival rates [42,64,65].

Further studies should correlate the biological performance of biomaterials with different viscoelastic properties, hydrophilicity, physicochemical differences and surface roughness, in order to better explore the impact of these properties in promoting osteoconductivity and long-term volume stability in bone regenerative therapy. Such properties are influenced by the production process and origin of the biomaterial and should be considered as requirements in regenerative procedures [6,42,57].

## 6. Conclusions

The sintering temperature affects the surface properties of chemically-deproteinized BHA biomaterials and these parameters influence the in vivo performance in terms of new bone formation and BMC. Non-sintered BHA or BHA sintered at low temperature (820 °C) seem to maintain a certain surface roughness of chemically-deproteinized BHA, allowing better bone regeneration and osteoconductivity (BMC). By contrast, sintering of BHA at 1200 °C has an effect on its morphological and structural characteristics and significantly modifies its biological performance (osteoconductivity) and crystallinity. Despite the presented results being only obtained on chemically deproteinized BHA, they tend to reemphasize the importance of the production process on the in vivo performances of bone regeneration biomaterials. The osteointegration of each particle of BHA plays an important role on the quality of the regenerated bone. Clinicians should take into account that the process of biomaterial manufacturing can significantly influence the degree of bone regeneration, when choosing their biomaterial for regenerative treatments.

## Figures and Tables

**Table 1:** Surface area, pore size and pore volume results.

	HAN	HA820	HA1200
Surface area (m^2^/g)	~36	~4.27	~0.27
Pore size (nm)	~10	~5.2	~4.5
Total pore volume (cm^3^/g)	~0.13	~0.007	~0.003
